# Tracing the evolution of the ZAR1 resistosome back to the Jurassic era

**DOI:** 10.1093/plcell/koad193

**Published:** 2023-07-04

**Authors:** Ching Chan

**Affiliations:** Assistant Features Editor, The Plant Cell, American Society of Plant Biologists, Rockville, MD, USA; Department of Life Science, National Taiwan Normal University, Taipei 11677, Taiwan

The R protein HOPZ-ACTIVATED RESISTANCE 1 (ZAR1) is a well-conserved coiled-coil (CC) domain-containing nucleotide-binding leucine-rich repeat receptor (NLR). ZAR1 was first identified in Arabidopsis as being required for recognition of the bacterial effector HopZ1a (Lewis et al. 2010). However, the range of effectors recognized by ZAR1 was subsequently shown to be quite broad, with this breadth enabled by an indirect recognition mechanism involving physical interactions with receptor-like cytoplasmic kinases (RLCKs), including RESISTANCE-RELATED KINASE 1 (RKS1) and PBS1-LIKE PROTEIN 2 (PBL2) (Breit-McNally et al. 2022). Structural analyses of the ZAR1-RKS1-PBL2 complex revealed a pentameric resistosome structure (Wang et al. 2019), which functions as a calcium channel specifically under pathogen infection (Bi et al. 2021). These exciting discoveries draw obvious attention to tracing the evolutionary history of the ZAR1 resistosome. **Adachi and colleagues** (Adachi et al. 2020; initially posted to bioRxiv in 2020) carried out a comprehensive phylogenetic analysis using 120 ZAR1 orthologs across 88 plant species, concluding that ZAR1 arose in early-flowering plant lineages during the Jurassic period ∼220 to 150 million years ago (see Fig.). Notably, this evolutionary history was independently confirmed in a concurrent study by Gong et al. (2022). These 2 back-to-back reports highlight ZAR1 as an ancient CC-NLR (alternatively CNL), which is quite unusual within the broader NLR gene family, especially those that bind directly to pathogen effectors.

**Figure.**
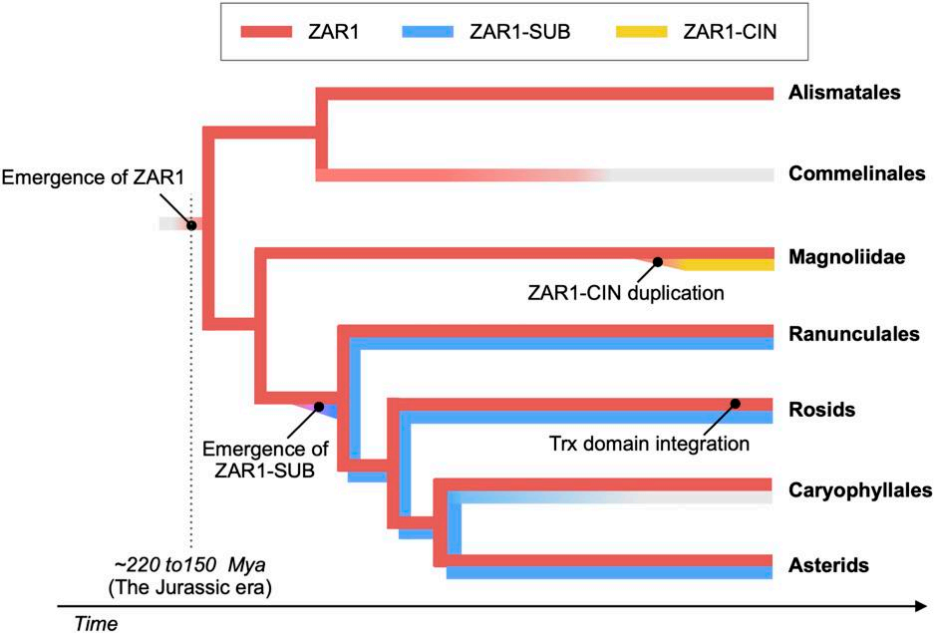
Evolution of ZAR1 genes in angiosperms. ZAR1 emerged ∼220 to 150 million years ago before the divergence of monocot and eudicot lineages. ZAR1-SUB emerged in eudicot evolution from a single ZAR1 duplication event. ZAR1-CIN emerged from segmental duplication and expansion of the ancestral ZAR1 gene in the Magnoliid species. Trx domain integration occurred in a few Rosids lineages. Adapted from Adachi et al. (2020) Figure 10A.

The above phylogenetic analyses revealed 3 distinct lineages of ZAR1-like genes: ZAR1, ZAR1-SUB, and ZAR1-CIN. A common feature for ZAR1 among distantly related plant species is the conservation of interaction sites with partner RLCKs, such as ZED1-related kinases (ZRKs). Conserved sequence patterns were annotated in critical regions for ZAR1 resistosome function, including the P-loop and MHD motifs for the binding and hydrolysis of dATP, the ZAR1-RLCK interface, and the MADA motif that activates cell death signaling. By heterologous expressing ZAR1 and ZRK orthologs in *Nicotiana benthamiana*, the authors observed autoactive cell death by introducing D to V mutation in some ZAR1 orthologs. Interestingly, in the case of stout camphor, CmZAR1^D488V^ was not autoactive by itself but triggered cell death when CmZRKs were coexpressed. These observations demonstrated the conservation of ZAR1 function and the functional connection between ZAR1 and ZRKs over the course of evolution in angiosperm. Notably, ZAR1 orthologs in cassava and cotton carry an integrated domain (ID) at their C termini. The C-terminal extensions were annotated to code for thioredoxin-like (Trx) domains. Although Gong et al. (2022) speculated that these might be annotation errors, Adachi et al. identified single reads spanning the domain ends in RNA-seq samples. More interestingly, these reads were found in specific tissues that were subjected to pathogen infection. Therefore, ZAR1-IDs are splicing variants representing a new NLR domain architecture specific to plant immunity. The ZAR1-SUB and ZAR1-CIN lineages are more diverged than the ZAR1 lineage. The sequence divergence was higher, and, in some cases, specific motifs were completely missing. Whether these structural variations lead to different interacting partners or effector recognition remains to be addressed. For instance, the ZAR1-SUB lineage was only identified in eudicots (although the Brassicales species generally have ZAR1 but lack ZAR1-SUB genes).

ZAR1 is no doubt one of the most important guardian receptors in plant innate immunity based on its ancient origin, wide distribution, and broad effector recognition capacity. However, the functional diversity of ZAR1 orthologs among different plant species remains largely unexplored. By investigating the evolutionary perspective of ZAR1, datasets presented by Adachi et al. (2020) and Gong et al. (2022) set the stage for further investigation in terms of ZAR1 structural variation, splicing control, functional redundancy, tissue-specific distribution, interaction partners, and the specificity of effector recognition.
